# Changes in child abuse experience associated to sleep quality: results of the Korean Children & Youth Panel Survey

**DOI:** 10.1186/s12889-021-11309-3

**Published:** 2021-06-24

**Authors:** Wonjeong Chae, Jieun Jang, Eun-Cheol Park, Sung-In Jang

**Affiliations:** 1 BK21 FOUR R&E Center for Precision Public Health, College of Health Science, Korea University, Seoul, Republic of Korea; 2 Institute of Health Services Research, Yonsei University, Seoul, Republic of Korea; 3 Department of Preventive Medicine, College of Medicine, Yonsei University, Seoul, Republic of Korea

**Keywords:** Child abuse, Sleep quality, Psychological development, Adolescence, Traumatic event, Child protection

## Abstract

**Background:**

A victim of child abuse can often develop mental illness. The early detection of mental illness of children could be supported by observing sleep quality. Therefore, we examined the relationship between sleep quality and the changes in child abuse by the child’s own parents over the study period.

**Methods:**

Data from the 2011–2013 Korean Children and Youth Panel Survey was used, and 2012 was set as the baseline. Adolescents who had poor sleep quality in 2011 were excluded from the analysis to obtain the final study population of 1276 adolescents aged 14 and 15 years. The generalized estimating equation model (GEE) was used for statistical analysis.

**Results:**

Children who had experienced and/or were currently experiencing child abuse showed significantly poorer sleep quality (current year abuse only: odds ratio [OR] = 0.57, 95% confidence interval [CI] = 0.41, 0.79; prior year abuse only: OR = 0.72, 95% CI = 0.52, 0.99; continuous abuse: OR = 0.56, 95% CI = 0.39, 0.80) compared to children who had no experience of child abuse.

**Conclusion:**

Child abuse remains a traumatic experience that influences the quality of sleep and hinders the child’s proper psychological development. We suggest approaching this issue at both the community and national levels to protect the victims.

**Supplementary Information:**

The online version contains supplementary material available at 10.1186/s12889-021-11309-3.

## Introduction

Child maltreatment is a universal problem that causes severe damage to children, with long-term consequences. The increasing number of reports of child maltreatment has become a major concern for children’s well-being [1, 2]. Child maltreatment occurs in the forms of child abuse and neglect of those younger than 18 years [3]. When broken down further, child abuse can be classified into physical abuse, sexual abuse, emotional or psychological abuse, and abandonment [1, 3, 4]. Attention to child maltreatment is rising in South Korea, with the proportion of cases reported to official agencies have increased noticeably. The reporting system reformed that social workers, doctors or teachers must report the abuse when the abuse is suspected. In 2015, there were a total of 11,715 cases reported to the National Child Protection Agency, compared to only 2105 in 2001 [5]. These cases comprised 37.7% physical abuse, 40.7% emotional or psychological abuse, 3.6% sexual abuse, and 18.0% neglect or abandonment. The highest rate appeared in the school children age between 7 to 15 years [5]. This group accounted for 62.6%, and the age group of 13 to 14 years was 23.0% of cases, which was the highest age group [5]. In the Child Maltreatment 2015 report by the Children’s Bureau of the United States, it was estimated that there are 4 million children who have experienced maltreatment. The highest number of child maltreatment occurred in children younger than 3 years [6]. The childhood memory of abuse continues to be a traumatic event, even into adulthood [1, 7, 8]. Children exposed to even a single type of maltreatment are more prone to high-risk and unhealthy behaviors [1, 5, 6].

The World Health Organization defines health in three dimensions, namely complete physical, mental, and social well-being [9]. Sleep quality is directly related to health and functioning [2, 10, 11], and it is important for both adults and adolescents. Poor sleep can cause negative effects on health by leading to sleep disorders, such as insomnia and sleep apnea, as well as other chronic health problems such as obesity, arthritis, diabetes, stroke, and immune system disorders [2, 12–14]. The relationships between sleep, cognitive function and mental health have been demonstrated for many years in previous studies on depression, and mood disorders [10, 12, 15–19]. Lack of sleep causes malfunction. Moreover, associations with dangerous health and social behaviors, including drug and alcohol use, interpersonal issues, suicidal thoughts, and violence, have been reported [10]. Sleep quality can influence both social behavior and daily life [2, 15, 17]. The mentioned health problems are critically linked to adolescents’ development and will appear in adulthood even if it is not currently exhibited.

One factor that causes sleep complaints or discomforts is traumatic events [14, 20–23]. Especially in children and adolescents, abused experience leads to poor sleep quality transitioning from childhood to adulthood; child abuse leaves a huge impact on their lives [1, 7, 19, 23, 24]. Child abuse from their own parents is generally not an acute trauma but instead occurs chronically and negatively influences their development [24]. Many previous studies on child abuse focused on its effects on physical, and mental health issues in adulthood with sleep problems [7, 9, 22, 24, 25]. Those studies show consistent findings that child abuse victims have suffered in their daily living, and it negatively influenced health behaviors. More specifically, studies by Bader K et al. reported an association between insomnia in adulthood and childhood neglect and abuse experience [26]. Noll JG et al. found sexual abused children experience can lead to sleep problems [27]. Libby AM et al. examined the two types of childhood abuse, physical and sexual, and their relationship to depressive and anxiety disorders [28]. There are limited studies conducted to seek association between child abuse and sleep quality concurrently and comparing child abuse status to the prior year.

The correlation among child abuse, sleep quality, and psychiatric illness in long term effects were well explained. Also, Turner S et al. studied the association between child maltreatment and sleep problems that prevent child maltreatment related to improving the child’s health outcome and well-being [11]. Moreover, studies discovered sleep and child abuse association that sleep could use as an indicator to measure and detect child abuse [2, 29]. Sleep can be measured in its quality and duration that different sleep patterns could be different in abused children [2]. As sleep and mental health are proven to be related, observing abused children’s sleep quality is needed [30, 31].

This study aimed to focus on the children who are experiencing or experienced abuse by their own parents in recent years (current year and prior year) and its effect on those children’s sleep quality. The study utilized a longitudinal study design to examine the effects of child abuse on sleep quality by examining the relationships between changes in child abuse year-to-year during the study period and sleep quality.

## Methods

### Study population

This study was conducted using data from the Korean Children & Youth Panel Survey (KCYPS), conducted by the National Youth Policy Institute. We analyzed 3 waves of the survey (2011–2013). The KCYPS is a longitudinal survey that is representative of Korean youth. The survey is conducted to assess the growth and development of children and youth at both the individual development and environmental levels, along with the influencing factors. The survey started in 2010 among first grade elementary school students, fourth grade elementary school students, and first grade middle school students. The sample was selected through stratified multi-stage clustering and random sampling from 16 administrative districts. Within those 16 administrative districts, survey participants were interviewed through the Tablet Assisted Personal Interview method.

Our target study population in this study was first-year middle school students who entered the survey in 2011 at the age of 13 years and who were followed until 2013, aged 15 years. A baseline study population comprising 1276 students was selected for the analysis after eliminating 521 students who reported experiencing poor sleep quality in 2011 or who had missing values for sleep-related questions. The elimination allowed our population to be set with adolescents who did not have sleep problems that the study was able to investigate adolescent’s sleep quality associated with changes in abuse experience.

### Outcome variables

**Fig. 1:**
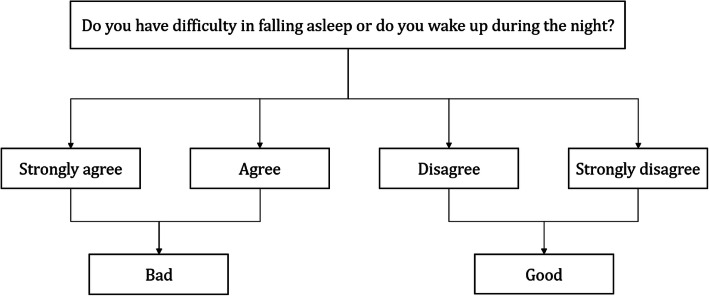
Sleep quality measure based on survey responses

The main outcome variables in the present study were sleep quality and sleep duration. Sleep quality is reported each year and was measured by the question, “Do you have difficulty in falling asleep or do you wake up during the night?”, with the following options “strongly agree,” “agree,” “disagree,” and “strongly disagree.” The responses were grouped into “good” (“disagree” and “strongly disagree”) and “bad” (“agree” and “strongly agree”) to indicate sleep quality (Fig. 1). Sleep duration was calculated by the question, “What time did you go to sleep and get up on average on weekdays (Monday-Friday) this semester?”

### Changes in child abuse experience year-to-year

**Fig. 2:**
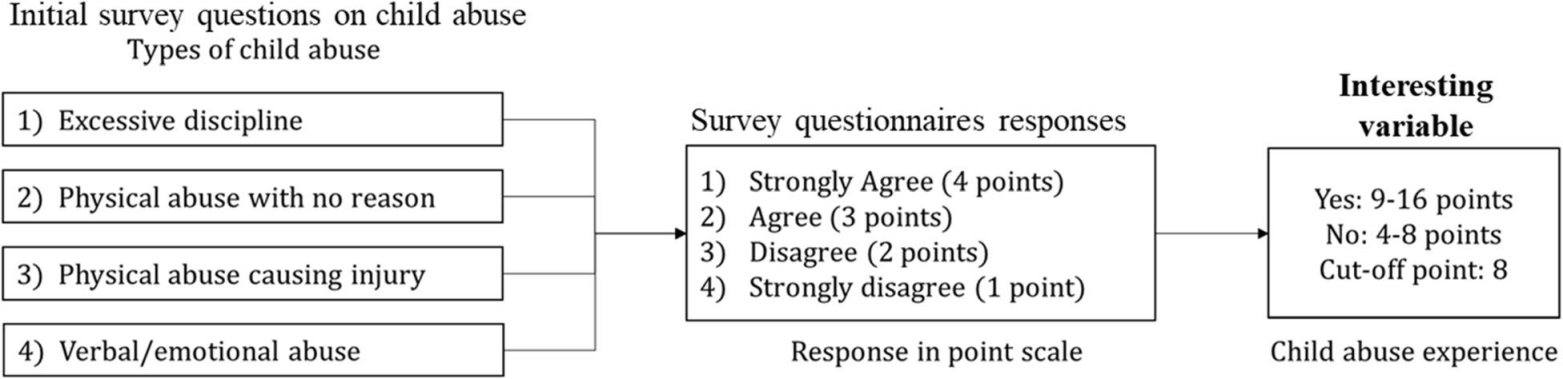
Child abuse experience based on survey responses

In order to obtain the incidences of child abuse experience, there were four questionnaires regarding the experience of “excessive discipline,” “physical abuse with no reason,” “physical abuse causing injury,” and “verbal/emotional abuse” by their own parents in the current year. For this study, four possible responses were rated on a 4-point scale from 1 to 4, corresponding to “strongly agree,” “agree,” “disagree,” and “strongly disagree” (Fig. 2). Using a combination of those responses, we set the cutoff score for child abuse experience at 8. If the score was 8 or above, it was considered that the child had experienced abuse because it represents the participant did not experience any abuse from their parents. So, 8 was set as the cutoff point.

To examine the changes in child abuse experience year-to-year during the study period, we created 4 categories, as follows: no—no (no experience of child abuse in the prior year and current year), no—yes (no experience of child abuse in the prior year, but experience of child abuse the current year), yes—no (experienced child abuse in the prior year, but no experience of child abuse the current year), and yes—yes (experienced child abuse in the prior year and current year).

### Covariates

For the study, variables focusing on the child and family were chosen. Child-related variables included sex (male or female), residency region (capital city, metropolitan area, or others), academic record (low, middle, or high), perceived health status (good or bad), depressive symptoms (yes or no), and mobile phone addiction score (low, middle, or high). Family-related variables included household income level in quartiles and the education level of the parents: high school graduate/below or college graduate/above. Further, the year (2012 or 2013) was also adjusted for in this study. All covariates were collected from self-reported survey questionnaires and responses.

### Statistical analysis

To observe the population’s general characteristics according to sleep quality and sleep duration, we used chi-square tests, t-test, and ANOVA. The variance inflation factors (VIFs) test was conducted for the variable selection, and all variables had resulted in less than 10, indicating there is no correlation between independent variables. To evaluate the association of changes in child abuse experiences with sleep quality and sleep duration during the study period, a generalized estimating equation model was used to measure repeated data using the statistical program SAS 9.4. All statistical tests were two-sided, and a *p-value* < .0.05 was considered statistically significant.

## Results

### General characteristics of the study population

The general baseline characteristics of the study population in 2012 according to the sleep quality and sleep duration, and divided by the changes in the child abuse experience year-to-year, are presented in Table 1.

**Table 1:** General characteristics according to sleep quality in 2012

Variables		Total		Sleep quality				***P***-value	Sleep duration		***P***-value
				Good		Poor					
		N	%	N	%	N	%		Mean ± SD		
**Total**		1276		975	76.4	301	23.6		7.126	1.129	
**Changes in child abuse experience**								<.0001			0.4449
	No→No	815	63.9	657	80.6	158	19.4		7.294	0.946	
	No→Yes	167	13.1	114	68.3	53	31.7		7.373	1.019	
	Yes→No	166	13.0	122	73.5	44	26.5		7.495	0.880	
	Yes→Yes	128	10.0	82	64.1	46	35.9		7.388	1.067	
**Gender**								0.3618			<.0001
	Men	644	50.5	499	77.5	145	22.5		7.497	0.931	
	Women	632	49.5	476	75.3	156	24.7		7.180	0.967	
**Residency region**								0.9658			0.3062
	Capital city	282	22.1	214	75.9	68	24.1		7.233	1.001	
	Metropolitan areas	436	34.2	333	76.4	103	23.6		7.395	0.953	
	Others	558	43.7	428	76.7	130	23.3		7.351	0.946	
**Household income level**								0.083			0.0470
	Quartile 1 (lowest)	285	22.3	215	75.4	70	24.6		7.491	0.948	
	Quartile 2	345	27.0	250	72.5	95	27.5		7.442	0.967	
	Quartile 3	366	28.7	283	77.3	83	22.7		7.287	0.970	
	Quartile 4 (highest)	280	21.9	227	81.1	53	18.9		7.130	0.920	
**Academic record**								0.0035			<.0001
	Low	384	30.1	271	70.6	113	29.4		7.536	0.939	
	Middle	361	28.3	279	77.3	82	22.7		7.378	0.955	
	High	531	41.6	425	80.0	106	20.0		7.173	0.955	
**Father’s education**								0.0841			0.3805
	High school grade/lower	547	42.9	405	74.0	142	26.0		7.471	0.961	
	College grade/higher	729	57.1	570	78.2	159	21.8		7.242	0.952	
**Mother’s education**								0.0321			0.3010
	High school grade/lower	703	55.1	521	74.1	182	25.9		7.438	0.967	
	College grade/higher	573	44.9	454	79.2	119	20.8		7.219	0.943	
**Perceived health status**								0.0026			0.3855
	Good	1179	92.4	913	77.4	266	22.6		7.344	0.948	
	Bad	97	7.6	62	63.9	35	36.1		7.293	1.122	
**Depressive symptoms**								<.0001			0.5892
	Yes	513	40.2	320	62.4	193	37.6		7.336	0.915	
	No	763	59.8	655	85.8	108	14.2		7.346	1.029	
**Mobile phone addiction score**								<.0001			0.1071
	Low(0–17)	435	34.1	358	82.3	77	17.7		7.415	0.904	
	Middle(8–11)	427	33.5	335	78.5	92	21.5		7.334	0.902	
	High(12–21)	414	32.4	282	68.1	132	31.9		7.268	1.072	

A total of 1276 subjects were selected at baseline after eliminating children who had poor sleep quality in the prior year. Of these, 975 children (76.4%) reported having good sleep quality, and 301 children (23.6%) reported having poor sleep quality. The pattern of change in the child abuse experience during the study period showed a statistical association with sleep quality. Within this variable, children with no experience of child abuse in the prior year and the current year accounted for the biggest proportion, at 63.9%, while the remaining categories shared similar proportion sizes of between 10 to 13%. The child’s academic record, perceived health status, depressive symptoms, and mobile phone addiction score, as well as the mother’s education, showed statistically significant relationships with sleep quality. In terms of sleep duration, the mean sleep duration in the total study population was 7.126 h; the academic record showed a statistical association.

### Result of the main analysis

After adjustment for possible confounders, the results of the generalized linear model of sleep quality and sleep duration are shown on Table 2.

**Table 2:** Generalized linear model with sleep quality in 2012–2013

Variables	Sleep quality			Sleep duration		
	Adjusted OR	95%CI		β*	S.E	***P***-value
**Changes in child abuse experience**						
No→No	1.00			Ref.		
No→Yes	0.57	(0.41	0.79)	0.171	0.070	0.0155
Yes→No	0.72	(0.52	0.99)	0.183	0.061	0.0028
Yes→Yes	0.56	(0.39	0.80)	0.067	0.079	0.3924
**Gender**						
Men	1.00			Ref.		
Women	1.05	(0.82	1.35)	−0.259	0.045	<.0001
**Residency region**						
Capital city	1.00			Ref.		
Metropolitan areas	1.03	(0.75	1.41)	−0.039	0.061	0.5257
Others	0.92	(0.67	1.26)	0.009	0.060	0.8874
**Household income level**						
Quartile 1 (lowest)	1.00			Ref.		
Quartile 2	0.92	(0.67	1.25)	−0.028	0.062	0.6545
Quartile 3	1.10	(0.79	1.53)	−0.195	0.065	0.0029
Quartile 4 (highest)	1.28	(0.87	1.89)	−0.302	0.070	<.0001
**Academic record**						
Low	1.00			Ref.		
Middle	1.37	(1.05	1.78)	−0.013	0.051	0.7922
High	1.31	(1.00	1.73)	−0.178	0.053	0.0007
**Father’s education**						
High school grade/lower	1.00			Ref.		
College grade/higher	1.16	(0.84	1.60)	−0.099	0.060	0.0998
**Mother’s education**						
High school grade/lower	1.00			Ref.		
College grade/higher	1.02	(0.74	1.42)	−0.060	0.059	0.3093
**Perceived health status**						
Good	1.00			Ref.		
Bad	0.67	(0.46	0.98)	−0.010	0.087	0.9080
**Depressive symptoms**						
Yes	0.35	(0.28	0.44)	−0.045	0.045	0.3115
No	1.00			Ref.		
**Mobile phone addiction score**						
Low(0–17)	1.00			Ref.		
Middle(8–11)	0.91	(0.69	1.20)	−0.031	0.047	0.5093
High(12–21)	0.67	(0.50	0.90)	−0.001	0.057	0.9888
**Year**						
2012	1.00			Ref.		
2013	1.90	(1.55	2.34)	−1.041	0.034	<.0001

Children who had experienced and/or were experiencing child abuse were more likely to have poor sleep quality (no—yes: odds ratio [OR] = 0.57, 95% confidence interval [CI] = 0.41, 0.79; yes—no: OR = 0.72, 95% CI = 0.52, 0.99; yes—yes: OR = 0.56, 95% CI = 0.39, 0.80). Children with higher academic records had better sleep quality (middle: OR = 1.37, 94% CI = 1.05, 1.78; high: OR = 1.31, 95% CI = 1.00, 1.73). Children with poor perceived health status (OR = 0.67, 95% CI = 0.46, 0.98) and with depressive symptoms (OR = 1.31, 95% CI = 1.00, 1.73) had relatively poor sleep quality. In terms of the analysis results for sleep duration, there was no variable associated with the patterns of change in the child abuse experience during the study period.

### Subgroup analysis with parent’s education level, perceived health status, and depressive symptom

Stratified subgroup analyses by the parents’ education level, perceived health status, and depressive symptom are recorded in Table 3. Children who experienced child abuse had decreased sleep quality, regardless of the time point of child abuse. Children experiencing child abuse in the current year showed statistically significant results. Children experiencing child abuse in the current year, but not in prior year, and who had parents with lower educational levels showed significantly decreased sleep quality (father’s education: OR = 0.48, 95% CI = 0.30, 0.77; mother’s education: OR = 0.54, 95% CI = 0.35, 0.81). Similarly, among children experiencing child abuse in both the prior year and the current year and who had parents with a lower educational level, the sleep quality also decreased, with statistical significance (father’s education: OR = 0.55, 95% CI = 0.33, 0.92; mother’s education: OR = 0.57, 95% CI = 0.36, 0.91). Regarding the children’s perceived health status and depressive symptoms, in children experiencing child abuse in the current year, statistical associations were found with good perceived health status (no—yes: OR = 0.54, 95% CI = 0.39, 0.76; yes—yes OR = 0.56, 95% CI = 0.39, 0.81) and with depressive symptoms (no—yes: OR = 0.47, 95% CI = 0.30, 0.72; yes—yes OR = 0.63, 95% CI = 0.41, 0.97).

**Table 3:** Subgroup analysis of sleep quality with child abuse experience change in 2012–2013

Variables		Changes in child abuse experience									
		No→No	No→Yes			Yes→No			Yes→Yes		
		OR	OR	95% CI		OR	95% CI		OR	95% CI	
**Father’s education**											
	High school grade/lower	1.00	0.48	(0.30	0.77)	0.67	(0.43	1.05)	0.55	(0.33	0.92)
	College grade/higher	1.00	0.67	(0.43	1.06)	0.79	(0.50	1.24)	0.57	(0.35	0.94)
**Mother’s education**											
	High school grade/lower	1.00	0.54	(0.35	0.81)	0.60	(0.40	0.89)	0.57	(0.36	0.91)
	College grade/higher	1.00	0.63	(0.37	1.05)	0.94	(0.55	1.63)	0.53	(0.31	0.93)
**Perceived health status**											
	Good	1.00	0.54	(0.39	0.76)	0.76	(0.54	1.06)	0.56	(0.39	0.81)
	Bad	1.00	0.71	(0.20	2.55)	0.48	(0.15	1.51)	0.48	(0.14	1.69)
**Depressive symptoms**											
	Yes	1.00	0.47	(0.30	0.72)	0.64	(0.41	1.00)	0.63	(0.41	0.97)
	No	1.00	0.78	(0.45	1.32)	0.83	(0.51	1.34)	0.40	(0.23	0.70)
**Mobile phone addiction score**											
	Low(0–17)	1.00	0.43	(0.23	0.81)	0.67	(0.38	1.20)	0.32	(0.16	0.66)
	Middle(8–11)	1.00	0.62	(0.35	1.08)	0.99	(0.55	1.77)	0.49	(0.26	0.91)
	High(12–21)	1.00	0.62	(0.36	1.04)	0.56	(0.34	0.94)	0.70	(0.41	1.19)

* Adjusted for gender, residency region, household income level and academic level

## Discussion

This study aimed to discover the associations between different patterns in the changes of child abuse year-to-year and sleep quality among Korean adolescents aged 13 to 15 years. As a result, the sleep quality of children who experienced child abuse was found to be low. Children who were victims of child abuse in the current year were more likely to have lower sleep quality than victims of child abuse in the prior year but not in the current year. Thus, child abuse can be considered a chronic factor related to poor sleep quality in adolescents.

A considerably large number of children are suffering from child abuse, which can lead to insufficient sleep, consequently leading to an unhealthy state. In our study, children with child abuse had a poorer sleep quality compared to children without child abuse. We divided the pattern of change in child abuse experience year-to-year into two groups: children who had no experience of child abuse in the prior year and those who had the experience of child abuse. However, when a child whose parents had not abused in the prior year but experienced child abuse in the current year, the quality of sleep dropped by about half. On the contrary, among children who had been abused by parents in the prior year, but not in the current year, their sleep quality was still low but showed an improvement compared to those experiencing steady abuse by their parents.

From the subgroup analysis on the depressive symptom, children who had depressive symptoms showed the lowest sleep quality when they were exposed to child abuse for the first time. They did not experience child abuse in the prior year but experiencing it in the current year. In contrast, children who did not have depressive symptoms showed the lowest sleep quality when they were continuously exposed to child abuse. The results support previous studies on sleep quality, child abuse, and depression [27, 32]. Many studies reported that abused children show depression that depression was used as a child abuse symptom [2, 31, 33]. However, our results warn to watch out for children who did not have any depressive symptoms in the child abuse case. Based on our results, we interpreted that children exposed to child abuse for the first time could show depressive symptoms, leading to poor sleep quality. However, children exposed to child abuse for a longer time do not show depressive symptoms, but the impact of child abuse appears as poor sleep quality. Therefore, we should have multi-screening tools to detect child abuse.

We interpreted the results of our study to indicate that child abuse is a traumatic event for adolescences and that it impacted their sleep quality. As a child, abuse becomes a traumatic event [1, 34] that can trigger reduced sleep quality. Child abuse and poor sleep quality have negative effects on children; previous studies have demonstrated these relationships [7, 22, 24, 34, 35], and our results shared consistent findings. In our results, the sleep quality seemed to be improved when the abuse stopped; however, the event is no longer an acute trauma but rather becomes a chronic trauma in children continuously abused. Among our study population, those who experienced abuse in congestive years have the lowest sleep quality. For children who experienced abuse from their own parents, the child abuse experience remains a traumatic event that affects their sleep, even if they are no longer receiving abuse [7, 14, 20, 25].

Among abused children, sleep disruption and sleep disorders are found that their sleep quality is low [15, 16, 19]. Sleep quality is closely related to the early detection of child abuse and psychological development. Monitoring the sleep quality of abused children allows the prevention of developing psychological issues, which can potentially lead to mental illness [2, 22, 25]. Moreover, the abused child has a trauma that their sleep quality would be lower compared to a child without abuse. The effect of sleep is essential to maintaining good health. Without sufficient sleep, sleep disorders, depression, mood disorders, low self-esteem, and weight problems may ensue [10, 12, 13, 15–18, 21]. Through these problems, it becomes hard for abused children to adjust to their surroundings and develop unhealthy behaviors and outcomes [1, 6, 34].

Moreover, poor sleep quality influences psychological problems [36, 37], which hinder adolescents from achieving proper psychological development. Such cases show that abused children are more likely to suffer from depression, emotion regulation disorders, aggressive behaviors, post-traumatic stress disorder, and sleep disorders; these develop as they grow [5, 8, 22, 28, 34]. To a child, the experience of abuse can become embedded as a chronic stressor with consistent abuse or as an acute stressor after only a short-term experience. Regardless of its duration and stress level, the experience negatively affects the child.

Children with experience of abuse tend to develop sleep problems more frequently, which may continue in their adulthood [22, 27]. The sleep problem appeared regardless of abuse type. The study of Greenfield et al. reported the association between child abuse and higher risk of global sleep pathology, and they found abuse is more related to sleep quality than sleep quantity, similar to our study. Greenfield et al. and Noll et al. conducted analyses on sexually abused children and sleep disturbance that showed such an experience is integrated to sleep quality. The impact of poor sleep quality on health can trigger various health issues, including psychological issues in adolescents and adulthood [10, 15, 17, 19].

In order to help children in family abuse, protection should be provided. Furthermore, sleep quality could be considered as an indicator to diagnose medical and/or social problems. Once the child has access to effective support from their family and a secure environment, their sleep quality is expected to improve. In turn, this should improve health and encourage positive social behavior [16, 19]. However, it may be hard to change the parents’ behavior, even though this would be the best solution. In addition to family support, social support mediates between the experience of abuse and its consequences [38]. Many studies have demonstrated that social support can help children with an experience of abuse to feel protected [39]. These children also face additional stressors as a result of abuse, such as separation from their family, experiences in foster care, and life-long victimization [40]. Moreover, the government level of intervention should be reinforced. Child abuse is hard to detect, and the rate of reported child abuse has increased about five times between 2001 to 2015 in Korea [5]. The possible reason Korea and the United States different age patterns in child abuse is due to the report system. As child abuse reporting is not highly motivated in Korea, child abuse detection at an early age is difficult. Therefore, more cases are reported in school aged children and adolescents. The abuse experience remains a traumatic event in the long-term. Therefore, within the community, the public should pay attention to abused children and provide safe shelters. The government enacted the Act on Punishment of Child Abuse Crime in 2014, which has resulted in a huge increment in reported child abuse cases to national agencies in Korea [5]. There are many promotions and campaigns to advocate child abuse and child protection regulations and laws worldwide. The government should develop and implement detailed policies and regulations to prevent child abuse. To protect the victims of child abuse, such policies should have a practical approach and consistent support.

The study was conducted using longitudinal data comprising a large number of children selected for the survey panel. This design provided a strong validity to examine sleep quality and its causality. The use of random sampling by stratification at the national level also adds strength to the validity of this study. While previous studies focused on discovering the long-term effects of child abuse [1, 4, 7], we focused on the effects of child abuse shortly after the event. In addition, we applied a lag time effect to the child abuse experience to determine the effects of changes in the event. We set the baseline population after eliminating those who had poor sleep quality previously and analyzed cases of new-onset poor sleep quality. Furthermore, we analyzed the effects of child abuse on the quality of sleep and sleep duration. We concluded that sleep quality is more related to the incidence of child abuse than sleep duration.

Despite the strengths of this study, there are some limitations to consider. First, the data was collected via self-report, and there is hence a risk of bias occurring during the data collection. Also, the study population’s age is limited to 13–15 because there was no national survey regarding child abuse experience for younger aged children. Second, the measure of sleep quality was not based on a diagnosis of sleep disorders, claims data, or obtained through the scientific method. However, sleep quality is a highly subjective element by the individual, for which it is acceptable to utilize self-reported data in children and adolescents [35]. However, further investigations with a quantitative measure such as a sleep quality index or using clinically proven data are suggested. In addition, the study with further consideration with depression is suggested. Child abuse and sleep quality are both closely related to depression, that depression is an important confounder of this study. Third, in our study, we found that a cell phone addiction related to poor sleep quality, similar to in previous research [41]. As cell phone addiction is increasing in adolescents, we recommend conducting further studies to elucidate the relationship between child abuse and cell phone addiction and study the inverse relation, whether child abuse triggers excessive use of cell phones since we observed a non-significant tendency. Lastly, regarding the study design, the results were not able to explain causal relationships between variables. As the cross-sectional study with repeated measures, our investigation was to find the association. Also, there is a possibility that we did not include all potential confounders that we suggest for the further study to include potential confounders that we did not use and cohort study to discover child abuse impact on sleep quality.

The analyses of the relationships between the patterns of change in the child abuse experience year-to-year and sleep quality provided a consistent implication that the abuse by their parents affected the children negatively, regardless of whether it was discontinued.

## Supplementary Information

**Additional file 1.**
