# Supplementary material for: Changes in child abuse experience associated to sleep quality: results of the Korean Children & Youth Panel Survey
Source: BMC Public Health. 2021 Jun 24;21:1210. doi: 10.1186/s12889-021-11309-3 (PMC8223356; doi:10.1186/s12889-021-11309-3)
Supplement: Supplementary file 1 — Additional file 1. [file 12889_2021_11309_MOESM1_ESM.docx]

Changes in child abuse experience associated to sleep quality: Results of the Korean Children & Youth Panel Survey

Wonjeong Chae^a, b^, Jieun Jang^a, b^, Eun-Cheol Park^b, c^, Sung-In Jang^b, c*^

^a^ Department of Public Health, College of Medicine, Yonsei University, Seoul, Republic of Korea

^b^ Institute of Health Services Research, Yonsei University, Seoul, Republic of Korea

^c^ Department of Preventive Medicine, College of Medicine, Yonsei University Seoul, Republic of Korea

Corresponding author: Sung-In Jang, MD, PhD

Department of Preventive Medicine & Institute of Health Services Research,

College of Medicine Yonsei University

50 Yonsei-ro, Seodaemun-gu, Seoul 120-752, Republic of Korea

Tel: +82-2-2228-1862; email: [JANGSI@yuhs.ac](mailto:JANGSI@yuhs.ac); fax: +82-2-392-8133

**Authors Email Addresses:**

Wonjeong Chae: [wjchae0816@yuhs.ac](mailto:wjchae0816@yuhs.ac)

Jieun Jang: [jieun99@yuhs.ac](mailto:jieun99@yuhs.ac)

Eun-Cheol Park: [ecpark@yuhs.ac](mailto:ecpark@yuhs.ac)

| **Appendix 1. Subgroup analysis of sleep duration with child abuse experience change in 2012-2013.** | | | | | | | | | | | | | | |
| --- | --- | --- | --- | --- | --- | --- | --- | --- | --- | --- | --- | --- | --- | --- |
| **Variables** | | **Changes in child abuse experience** | | | | | | | | | | | | |
|  |  | **No→No** | | | **No→Yes** | | | **Yes→No** | | | | **Yes→Yes** | | |
|  |  | **β*** | **β*** | **S.E** | | ***P*-value** | **β*** | | **S.E** | ***P*-value** | **β*** | | **S.E** | ***P*-value** |
| **Father's education** | |  |  |  | |  |  | |  |  |  | |  |  |
|  | High school grade/lower | Ref. | 0.168 | 0.112 | | 0.1315 | 0.166 | | 0.092 | 0.0706 | 0.253 | | 0.122 | 0.0385 |
|  | College grade/higher | Ref. | 0.164 | 0.089 | | 0.0654 | 0.197 | | 0.083 | 0.0170 | -0.081 | | 0.098 | 0.4065 |
| **Mother's education** | |  |  |  | |  |  | |  |  |  | |  |  |
|  | High school grade/lower | Ref. | 0.162 | 0.095 | | 0.0869 | 0.209 | | 0.079 | 0.0084 | 0.238 | | 0.104 | 0.0216 |
|  | College grade/higher | Ref. | 0.184 | 0.103 | | 0.0755 | 0.149 | | 0.096 | 0.1235 | -0.137 | | 0.115 | 0.2332 |
| **Perceived health status** | |  |  |  | |  |  | |  |  |  | |  |  |
|  | Good | Ref. | 0.207 | 0.072 | | 0.0041 | 0.201 | | 0.064 | 0.0016 | 0.068 | | 0.081 | 0.3961 |
|  | Bad | Ref. | -0.196 | 0.265 | | 0.4589 | 0.033 | | 0.241 | 0.8918 | 0.029 | | 0.284 | 0.9178 |
| **Depressive symptoms** | |  |  |  | |  |  | |  |  |  | |  |  |
|  | Yes | Ref. | 0.183 | 0.108 | | 0.0908 | 0.126 | | 0.098 | 0.1975 | 0.043 | | 0.108 | 0.6935 |
|  | No | Ref. | 0.165 | 0.094 | | 0.0789 | 0.218 | | 0.078 | 0.0049 | 0.088 | | 0.116 | 0.4473 |
| **Mobile phone addiction score** | |  |  |  | |  |  | |  |  |  | |  |  |
|  | Low(0-17) | Ref. | 0.135 | 0.120 | | 0.2610 | 0.093 | | 0.105 | 0.3742 | -0.018 | | 0.146 | 0.9031 |
|  | Middle(8-11) | Ref. | 0.207 | 0.115 | | 0.0733 | 0.242 | | 0.099 | 0.0148 | -0.016 | | 0.103 | 0.8779 |
|  | High(12-21) | Ref. | 0.177 | 0.130 | | 0.1727 | 0.213 | | 0.113 | 0.0604 | 0.160 | | 0.140 | 0.2532 |

* Adjusted for gender, residency region, household income level and academic level.
